# Neuroimmune Responses in a New Experimental Animal Model of Cerebral Aspergillosis

**DOI:** 10.1128/mbio.02254-22

**Published:** 2022-08-30

**Authors:** Brianne N. Sullivan, Mia A. Baggett, Christa Guillory, MaryJane Jones, Chad Steele

**Affiliations:** a Department of Microbiology and Immunology, Tulane University, New Orleans, Louisiana, USA; b Neuroscience Program, Tulane Brain Institute, Tulane University, New Orleans, Louisiana, USA; Albert Einstein College of Medicine

**Keywords:** cerebral aspergillosis, immunosuppression, model, neuroimmune, neuroinflammation, *Aspergillus fumigatus*, central nervous system infections

## Abstract

Exposure of immunosuppressed individuals to the opportunistic fungal pathogen Aspergillus fumigatus may result in invasive pulmonary aspergillosis (IPA), which can lead to the development of cerebral aspergillosis (CA), a highly lethal infection localized in the central nervous system (CNS). There are no experimental models of CA that effectively mimic human disease, resulting in a considerable knowledge gap regarding mechanisms of neurological pathogenicity and neuroimmune responses during infection. In this report, immunosuppressed mice (via acute, high-dose corticosteroid administration) challenged with A. fumigatus resting conidia intranasally, followed a day later by a 70-fold lower inoculum of pre-swollen conidia intravenously (IN + IV + steroid), demonstrated increased weight loss, signs of severe clinical disease, increased fungal burden in the brain, and significant reduction in survival compared to immunosuppressed mice challenged intranasally only (IN + steroid) or non-immunosuppressed mice challenged both intranasally and intravenously (IN + IV). The IN + IV + steroid group demonstrated significant decreases in monocytes, eosinophils, dendritic cells (DCs), and invasive natural killer T (iNKT) cells, but not neutrophils or γδ T cells, in the brain compared to the IN + IV group. Likewise, the IN + IV + steroid group had significantly lower levels of interleukin (IL)-1β, IL-6, IL-17A, CC motif chemokine ligand 3 (CCL3), CXC chemokine ligand 10 (CXCL10), and vascular endothelial growth factor (VEGF) in the brain compared to the IN + IV group. IN + IV + steroid was superior to both IN + IV + chemotherapy (cytarabine + daunorubicin) and IN + IV + neutropenia for the development of CA. In conclusion, we have developed a well-defined, physiologically relevant model of disseminated CA in corticosteroid-induced immunosuppressed mice with a primary pulmonary infection. This model will serve to advance understanding of disease mechanisms, identify immunopathogenic processes, and help define the protective neuroinflammatory response to CA.

## INTRODUCTION

Invasive pulmonary aspergillosis (IPA), a disease most attributed to Aspergillus fumigatus, is a significant cause of illness and death in immunosuppressed patients. While A. fumigatus is readily eliminated from the lung in immunocompetent patients through innate immune clearance, immunosuppressed individuals often demonstrate impaired innate immune responses. At-risk populations include patients exposed to immunosuppressive therapies associated with hematologic malignancies (HMs), stem cell and solid-organ transplants, and chronic pulmonary diseases. Prolonged neutropenia, graft-versus-host disease, high-dose corticosteroid treatment, and other immunosuppressive regimens are considered the top risk factors. Worldwide, IPA is diagnosed in more than 300,000 immunocompromised patients annually (1, 2). During IPA, unchecked fungal growth results in tissue damage and potential dissemination to other organs. Disseminated disease occurs in 20 to 50% of the IPA population, and most frequently occurs via hematogenous spread from the lungs to subsequent organs (3, 4). The central nervous system (CNS) is often reported as the most frequent site of Aspergillus dissemination from the lung resulting in cerebral aspergillosis (CA) (4, 5). CA occurs in up to 40% of certain immunocompromised populations, averaging nearly 20% in all patients with disseminated aspergillosis (4, 6, 7).

In addition to being considered the most frequent site of secondary infection, CA is also deemed the most fatal, with mortality rates reaching 90% in specific patient populations (5). Symptoms of CA are wide-ranging and often nonspecific and include fever that is resistant to antibacterial treatment, headache, altered mental status, lethargy, nausea/vomiting, focal neurological deficits, and seizures (8). This wide range of symptoms is greatly due to the multiple potential clinical presentations of CA, such as meningitis, encephalitis, mycotic aneurysms, granuloma, cerebral blood vessel invasion with or without infarction, secondary infection, hemorrhage, and most frequently, single or multiple brain abscesses (6, 8, 9). Death from CA is often rapid, reportedly occurring in as few as 5 days postinfection, and is predominately associated with hemorrhagic and/or ischemic events (6, 9). While the continued development and implementation of anti-fungal drugs have greatly improved the outcome of IPA patients, very few available therapeutics are effective in the CNS for the treatment of CA.

Studies have identified distinct cellular recruitment and inflammatory mediator profiles involved in the innate immune response during A. fumigatus lung infection (10, 11). In contrast, the immune cell and inflammatory mediator profiles associated with A. fumigatus-driven CA have not been thoroughly investigated. Additionally, unlike for IPA, there are no *in vivo* which that effectively mimic the human disease, i.e., primary pulmonary infection with subsequent dissemination through the blood resulting in CA. To date, models of A. fumigatus brain infection have been conducted using non-physiologic intracranial or intravenous challenge and have primarily focused on antifungal drug efficacy or protection strategies (12–14). The development of a CA model with lung involvement under clinically relevant immunosuppression would result in a better understanding of immune responses in the CNS and provide a platform for future examination of therapeutics and diagnostics for CA. To this end, in this report we detail the development of a novel preclinical murine model of CA.

## RESULTS

### Development of an immunosuppression-based “two-hit” disseminated cerebral aspergillosis model.

**FIG 1:**
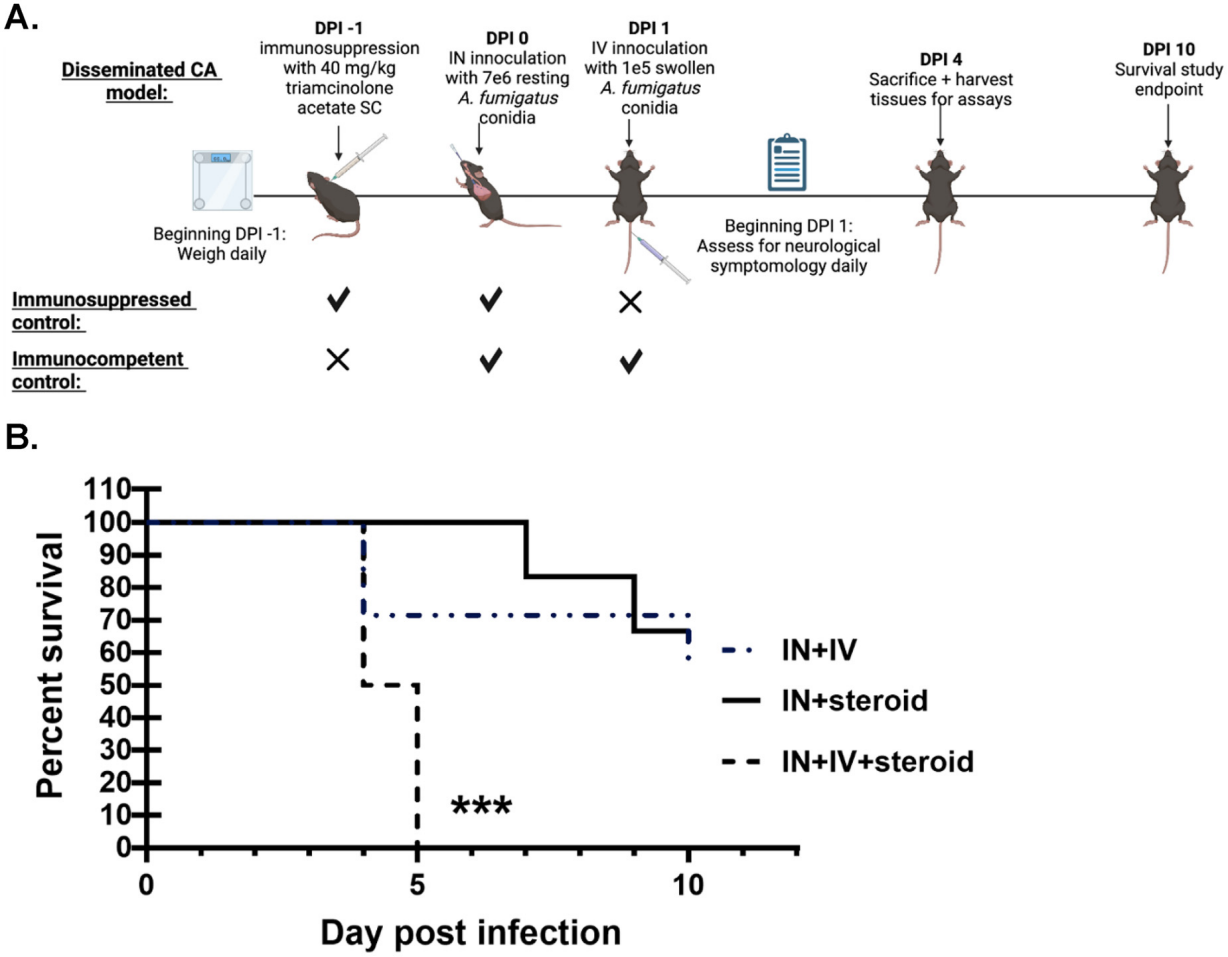
Development of an immunosuppression-based “two-hit” disseminated cerebral aspergillosis model. (A, B) C57BL/6 mice were immunosuppressed with triamcinolone acetate (Kenalog-40; 40 mg/kg) subcutaneously (SC) 1 day before IN inoculation with 7 × 10^6^ Aspergillus fumigatus resting conidia; mice were subsequently inoculated with 1 × 10^5^ A. fumigatus swollen conidia via the lateral tail vein (IV). For survival assessment, mice were monitored through day 10. Cumulative data from two independent studies (*n* = 3 to 4 mice per group per study). *, *P* = 0.026; ***, *P* = 0.0009 (graph created by Kaplan-Meier estimator, groups analyzed by Mantel-Cox log-rank test). Image created with BioRender.com.

To develop a clinically relevant disseminated CA model, C57BL/6 mice were immunosuppressed with corticosteroids, a known risk factor for the development of CA pursuant to IPA (9, 15). Immunosuppressed mice were challenged with A. fumigatus resting conidia intranasally, followed a day later by a 70-fold lower inoculum of pre-swollen conidia intravenously (IN + IV + steroid) (Fig. 1A). The rationale for employing swollen conidia is that these organisms are more metabolically and biochemically active compared to inhaled resting conidia and are more likely to escape from the lung and enter the bloodstream (16). Control groups included immunosuppressed mice challenged intranasally only (IN + steroid) and non-immunosuppressed mice challenged both intranasally and intravenously (IN + IV) (Fig. 1A). Immunosuppression followed by the IN + IV challenge resulted in a progressively lethal infection, with 50% of the mice succumbing to disease by day 4 and 100% by day 5 (Fig. 1B). In the IN + IV group, 40% of mice succumbed by day 10, whereas in the IN + steroid group, 30% of the mice succumbed by day 10. Thus, a “two-hit” model of A. fumigatus resting conidia administered IN followed a day later by administration of swollen conidia i.v. under corticosteroid-driven immunosuppression results in rapid mortality.

### Disease severity in an immunosuppression-based “two-hit” disseminated cerebral aspergillosis model.

**FIG 2:**
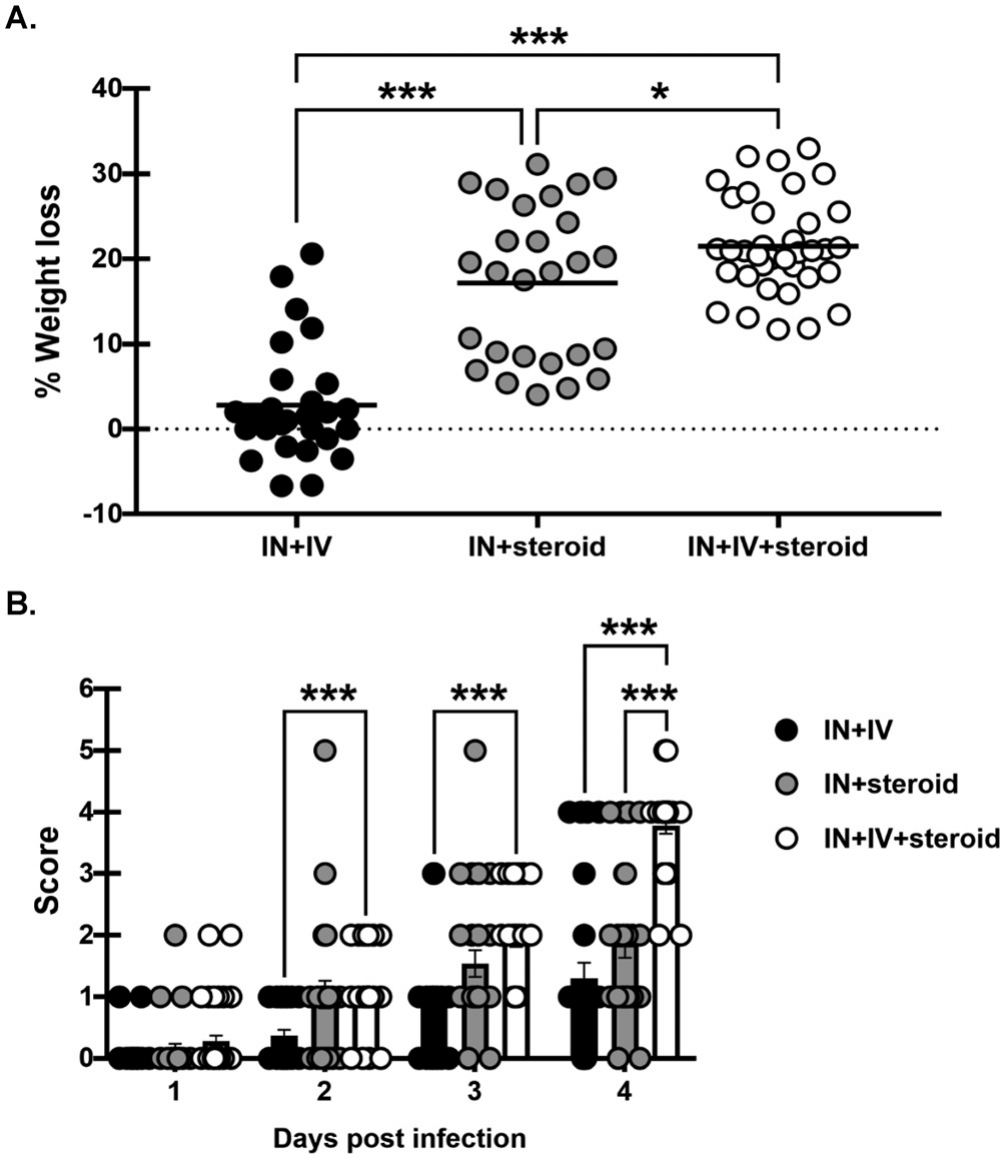
Disease severity in an immunosuppression-based “two-hit” disseminated cerebral aspergillosis model. Mice were immunosuppressed and exposed to A. fumigatus as in Fig. 1. (A) Total percentage of weight loss was calculated by comparing mouse weight at the study endpoint (day 4) with that at the study initiation (day −1). (B) Beginning on day 1, mice were assessed for disease severity and assigned a score between 0 and 5 correlating to signs of disease severity. Cumulative data from eight independent studies. For all graphs, * and *** represent *P* values of <0.05 and <0.001, respectively; *n* = 3 to 4 mice/group for each study; each data point/dot represents a single mouse; line in each group corresponds to the mean (panel A, unpaired two-tailed Student’s *t* test; panel B, two-way analysis of variance [ANOVA; mixed effects] with *post hoc* Tukey’s multiple-comparison test).

Because the IN + IV + steroid group had a significantly reduced survival rate early and overall in comparison to both control groups, day 4 post infection was chosen for additional analyses. Beginning 1 day after IN challenge, mice were monitored for weight loss and clinical disease signs through day 4. Death was recorded for mice who succumbed to the disease (a score of 5) and those which met criteria that necessitated humane euthanasia (score of 4). Overall, mice subjected to immunosuppression (IN + IV + steroid and IN + steroid) had significantly more weight loss than immunocompetent mice (IN + IV) (Fig. 2A). In line with mortality, mice in the IN + IV + steroid group lost a significantly greater percentage of total body weight in comparison to the other groups (Fig. 2A). Likewise, disease severity was also worse in the IN + IV + steroid group in comparison to the other groups (Fig. 2B). Thus, the “two-hit” disseminated CA model results in more substantial weight loss and increased clinical scores compared to mice with IPA alone, suggesting that these mice have more severe disease.

### Fungal burden in an immunosuppression-based “two-hit” disseminated cerebral aspergillosis model.

**FIG 3:**
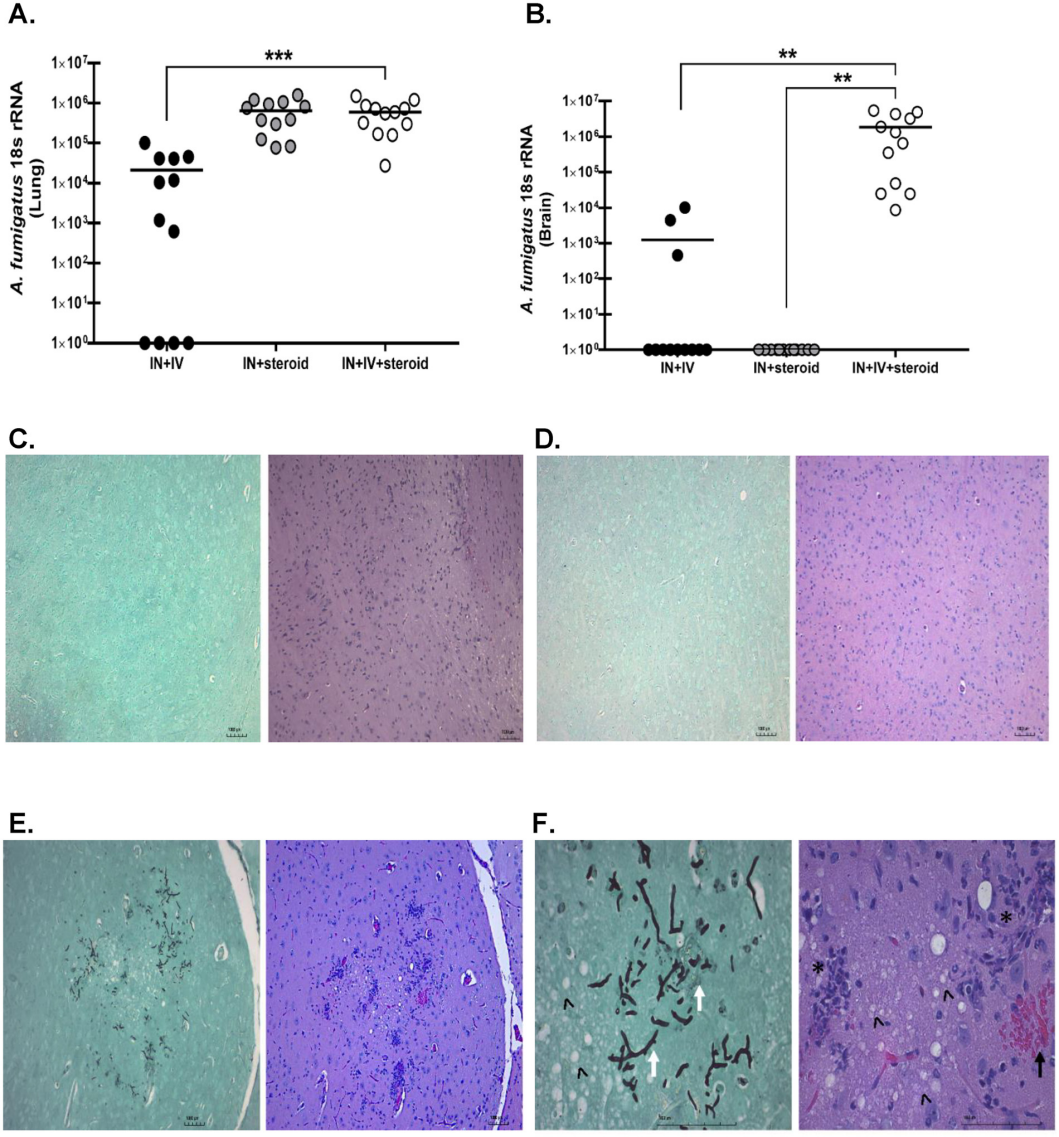
Fungal burden in an immunosuppression-based “two-hit” disseminated cerebral aspergillosis model. Mice were immunosuppressed and exposed to A. fumigatus as in Fig. 1. At day 4, half of the brain was collected and homogenized, and RNA was isolated. (A) Lung and (B) brain fungal burdens at day 4 were assessed by reverse transcription-quantitative PCR (RT-qPCR) analysis of A. fumigatus 18S rRNA levels. Cumulative data from three independent studies. For all graphs, ** indicates *P* < 0.01; *n* = 3 to 4 mice/group for each study; each data point represents a single mouse; and the line in each group corresponds to the mean (unpaired two-tailed Student’s *t* test). At day 4, one hemisphere was collected and fixed with 10% neutral buffered formalin, after which the tissue was paraffin-embedded, sectioned along the sagittal plain, and stained for histological analysis. (C to E) Representative Grocott’s methenamine silver (GMS)-stained brain sections (left images) from the brains of (C) IN + IV, (D) IN + steroid, and (E) IN + IV + steroid group mice. Original magnification = ×10. Scale bar = 1,000 μm. Representative hematoxylin and eosin (H&E)-stained brain sections (right images) from the brains of (C) IN + IV, (D) IN + steroid, and (E) IN + IV + steroid group mice. Original magnification = ×10. Scale bar = 1,000 μm. (F) Representative GMS (left) and H&E (right) images from the same slide as in panel E. Original magnification = ×40. Scale bar = 1,000 μm. White arrows indicate representative fungal hyphae, ˄ indicate areas of necrosis, * indicates cell infiltration, and black arrows indicate hemorrhage.

Human IPA and subsequent dissemination to the CNS is associated with fungal infiltration and germination at the infection sites. As expected, all mice had detectable A. fumigatus in the lung, with both immunosuppressed groups (IN + IV + steroid and IN + steroid) having higher fungal burdens than the immunocompetent group (IN + IV) (Fig. 3A). Unexpectedly however, despite the presence of immunosuppression, mice in the IN + steroid group did not have detectable A. fumigatus in the brain (Fig. 3B). Likewise, only 25% of the mice in the IN + IV group were positive for A. fumigatus in the CNS (Fig. 3B). In contrast, A. fumigatus was observed in the brain in 100% of the mice in the IN + IV + steroid group (Fig. 3B). Histopathology of the CNS in patients with CA often demonstrates necrosis, cellular infiltrates, and fungal organisms (17, 18). Hematoxylin and eosin (H&E) and Grocott’s methenamine silver (GMS)-stained sagittal brain sections demonstrated no remarkable features in the immunocompetent (IN + IV) (Fig. 3C) and immunosuppressed (IN + steroid) groups (Fig. 3D). Conversely, fungal organisms and pathological changes were observed in the brains of mice with disseminated CA (IN + IV + steroid) (Fig. 3E). Specifically, fungal hyphae, necrosis, cellular infiltrates, and hemorrhage were readily observed in mice from the IN + IV + steroid group (Fig. 3F). Thus, immunosuppression is a prerequisite for the development of CA after IV administration of swollen conidia 1 day after IN with resting conidia. Moreover, the presence of specific pathological features further indicates that the disseminated CA model successfully recapitulates key aspects of the human disease.

### Immune cell infiltration into the brain in an immunosuppression-based “two-hit” disseminated cerebral aspergillosis model.

**FIG 4:**
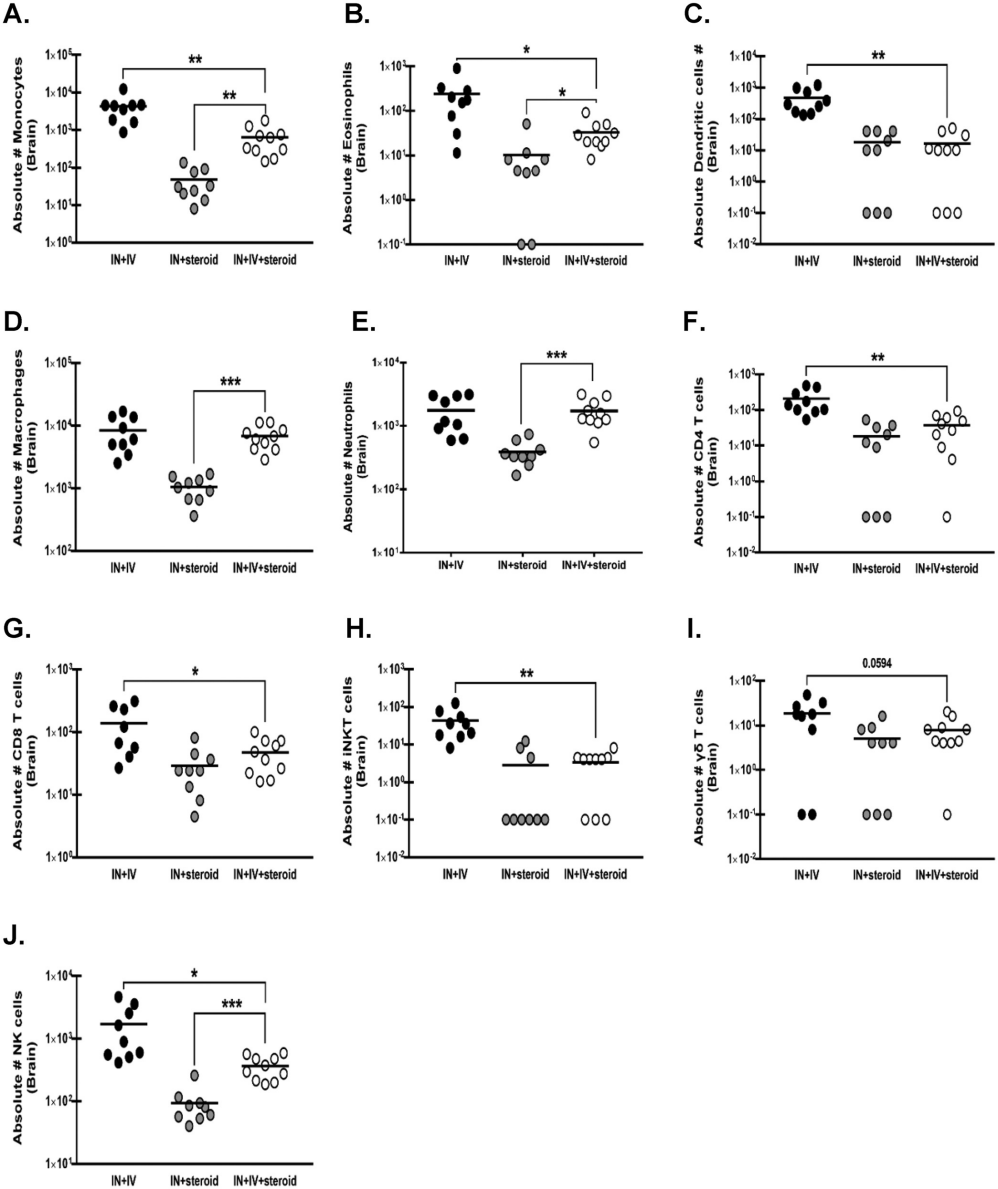
Immune cell infiltration into the brain in an immunosuppression-based “two-hit” disseminated cerebral aspergillosis model. Mice were immunosuppressed and exposed to A. fumigatus as in Fig. 1. On day 4, whole brains were collected and mononuclear cells were isolated following enzymatic digestion and quantified by flow cytometry. (A) Monocytes (CD45^+^ CD11b^+^ CD11c^−^ Ly6C^+^), (B) eosinophils (CD45^+^ CD11b^+^ Siglec F^+^), (C) dendritic cells, (CD45^+^ CD11c^+^ Ly6C^+^ MHC II^+^), (D) macrophages (CD45hiCD11b^+^ TMEM119-), (E) neutrophils (CD45^+^ CD11b^+^ Ly6G^+^), (F) CD4 T cells (CD45^+^ CD3^+^ CD4^+^), (G) CD8 T cells (CD45^+^ CD3^+^ CD8^+^), (H) invasive natural killer T (iNKT) cells (CD45^+^ CD3^+^ CD1d^+^), (I) γδ T cells (CD45^+^ CD3^+^ TCRγδ^+^) and (J) natural killer (NK) cells (CD45^+^ CD3^−^ NK1.1^+^). Cumulative data from three independent studies (*n* = 3 to 4 mice per group per study). Each data point represents an individual sample. Line within a given group represents the mean. For all graphs, *, **, and *** represent *P* values of <0.05, <0.01, and <0.001, respectively (unpaired two-tailed Student’s *t* test).

Cells of the myeloid and lymphoid lineage are vital for the immune response to A. fumigatus in the lung; however, their response within the brain has not been characterized (19, 20). Immune cell suppression by corticosteroids is well-documented as a contributor to the development of severe disease during IPA (21). Compared to the immunocompetent control mice (IN + IV), mice in the disseminated CA model (IN + IV + steroid) had significantly lower inflammatory monocyte (Fig. 4A), eosinophil (Fig. 4B), and dendritic cell (DC) counts (Fig. 4C) in the brain. However, in comparison to the immunosuppressed control mice (IN + steroid), the number of monocytes, eosinophils, macrophages (Fig. 4D), and neutrophils (Fig. 4E) was significantly elevated in the brain of disseminated CA model mice, presumably as a result of the higher fungal burden in these mice. Comparatively, most lymphoid lineage cells were affected by corticosteroid immunosuppression. The numbers of CD4 (Fig. 4F), CD8 (Fig. 4G), invasive natural killer T (iNKT) (Fig. 4H), γδ T cells (Fig. 4I), and natural killer (NK) cells (Fig. 4J) were all lower in the brains of the IN + IV + steroid group than in those of the IN + IV group. The numbers and activation states of primary resident immune mediators in the brain, microglia, and astrocyte subsets were unchanged in the brains of immunocompetent versus immunosuppressed control mice (Fig. S1A to F). Thus, despite the presence of germinating organisms in the brain, immune cell infiltration into the CNS was dramatically impaired as a result of corticosteroid-induced immunosuppression.

10.1128/mbio.02254-22.1 FIG S1 Neuroimmune cell populations were unaffected in the brain of “two-hit” disseminated CA model mice. Mice were immunosuppressed and exposed to Aspergillus fumigatus as in Fig. 1. At day 4, whole brains were collected, mononuclear cells isolated following enzymatic digestion and quantified by flow cytometry. (A) Microglia (CD45intCD11b^+^ TMEM119^+^); (B) M1 microglia (CD45intCD11b^+^ TMEM119^+^ CD86^+^ CD206^−^), (C) M2 microglia (CD45intCD11b^+^ TMEM119^+^ CD86^−^ CD206^+^), (D) astrocytes (CD45^−^ CD11b^−^ ASCA-2^+^ A2B5^+^), and (E) reactive astrocytes (CD45^−^ CD11b^−^ ASCA-2^+^ A2B5^+^ GFAP^+^) were quantified by flow cytometry. Cumulative data from three independent studies (*n* = 3 to 4 mice per group per study). Each data point represents an individual sample. The line within a given group represents the mean. Download FIG S1, TIF file, 0.2 MB. Copyright © 2022 Sullivan et al. 2022 Sullivan et al. https://creativecommons.org/licenses/by/4.0/ This content is distributed under the terms of the Creative Commons Attribution 4.0 International license.

### Inflammatory cytokine and chemokine responses in an immunosuppression-based “two-hit” disseminated cerebral aspergillosis model.

**FIG 5:**
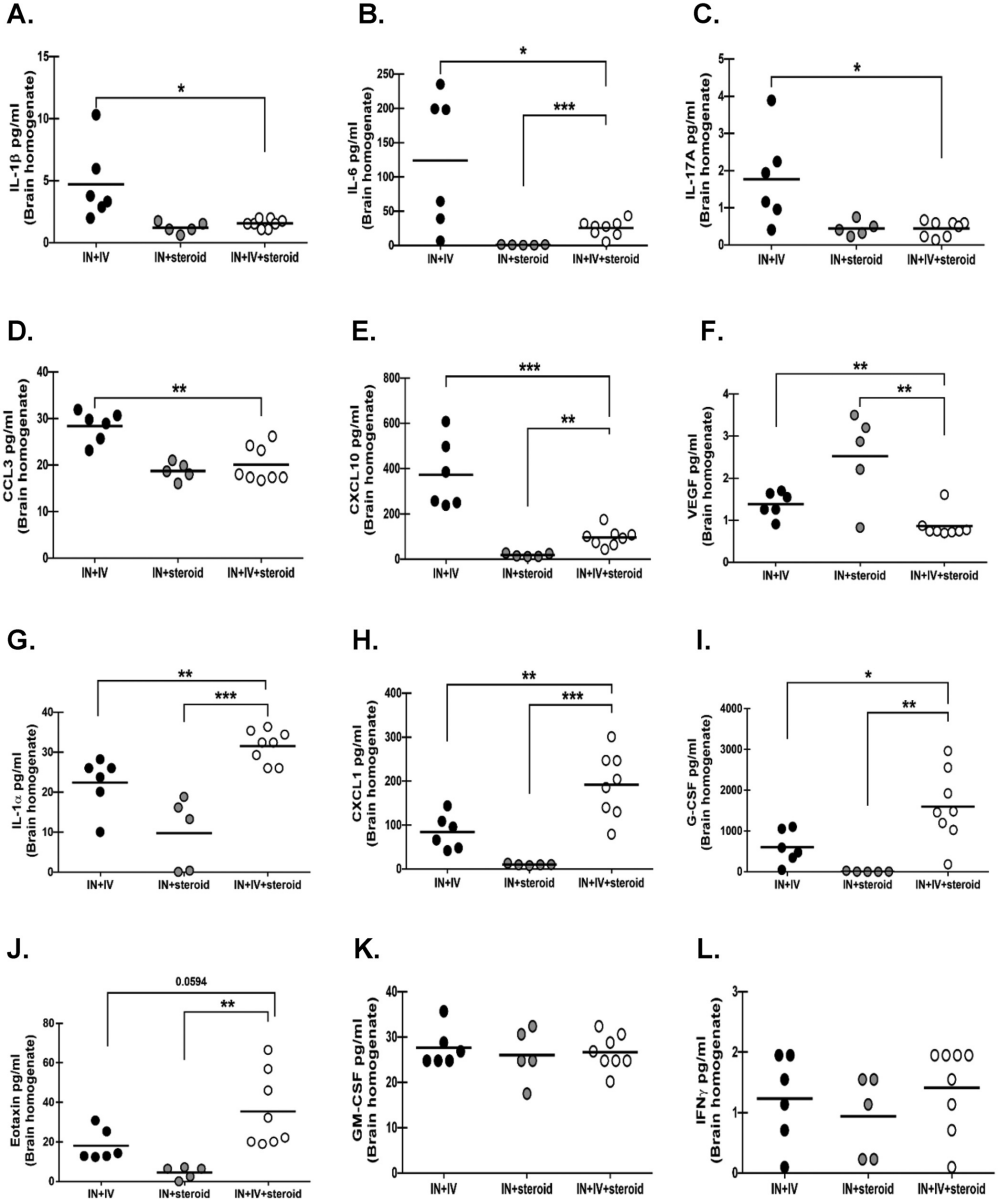
Inflammatory cytokine and chemokine responses in an immunosuppression-based “two-hit” disseminated cerebral aspergillosis model. Mice were immunosuppressed and exposed to A. fumigatus as in Fig. 1. On day 4, half of the brain was collected and homogenized and supernatants were clarified. Levels of (A) interleukin (IL)-1β, (B) IL-6, (C) IL-17A, (D) CC motif chemokine ligand 3 (CCL3), (E) CXC chemokine ligand 10 (CXCL10), (F) vascular endothelial growth factor (VEGF), (G) IL-1α, (H) CXCL1, (I) granulocyte colony-stimulating factor (G-CSF), (J) eotaxin, (K) granulocyte-macrophage CSF (GM-CSF), and (L) IFN-γ were quantified in clarified brain homogenates by Luminex-based Milliplex analysis. Cumulative data from two independent studies (*n* = 3 to 4 mice per group per study). Each data point represents an individual sample. Line within a given group represents the mean. For all graphs, *, **, and *** represent *P* values of <0.05, <0.01, and <0.001, respectively (unpaired two-tailed Student’s *t* test).

Multiple inflammatory mediators, such as interleukin (IL)-1α, IL-1β, IL-6, tumor necrosis factor α, and IL-17A, are required for protection against IPA (22). Moreover, mediators such as granulocyte-macrophage colony-stimulating factor (GM-CSF; sargramostim/Leukine) have been approved to shorten the time of immunosuppression and reduce the incidence of infection associated with chemotherapy and bone marrow transplantation, whereas interferon gamma (IFN-γ; Actimmune) has been approved for the treatment of infectious fungal complications associated with chronic granulomatous disease (23–26). To gain insight into inflammatory mediator pathways that may be critical for defense in the CNS, we focused on the levels of mediators in the brain that were significantly different between the IN + IV + steroid group and the IN + IV group. First, it was interesting to note that despite only 25% of mice in the IN + IV group having detectable A. fumigatus in the brain, 100% of mice had detectable levels of multiple inflammatory mediators. This analysis revealed that IL-1β (Fig. 5A), IL-6 (Fig. 5B), IL-17A (Fig. 5C), CC motif chemokine ligand 3 (CCL3; Fig. 5D), CXC chemokine ligand 10 (CXCL10; Fig. 5E), and vascular endothelial growth factor (VEGF; Fig. 5F) were decreased in the IN + IV + steroid group despite the presence of higher fungal burdens. In contrast, mediators that were elevated in the IN + IV + steroid group included IL-1α (Fig. 5G), CXCL1 (Fig. 5H), granulocyte colony-stimulating factor (G-CSF; Fig. 5I), and eotaxin (Fig. 5J). Surprisingly, the mediators potentially used for the treatment of IPA, GM-CSF and IFN-γ, were not different between the groups (Fig. 5K and L). Thus, the mediators suppressed by corticosteroids versus those enhanced by elevated fungal burden may represent important CNS host defense pathways during CA.

### Comparison between corticosteroid-mediated immunosuppression and chemotherapy-mediated immunosuppression in the “two-hit” disseminated cerebral aspergillosis model.

**FIG 6:**
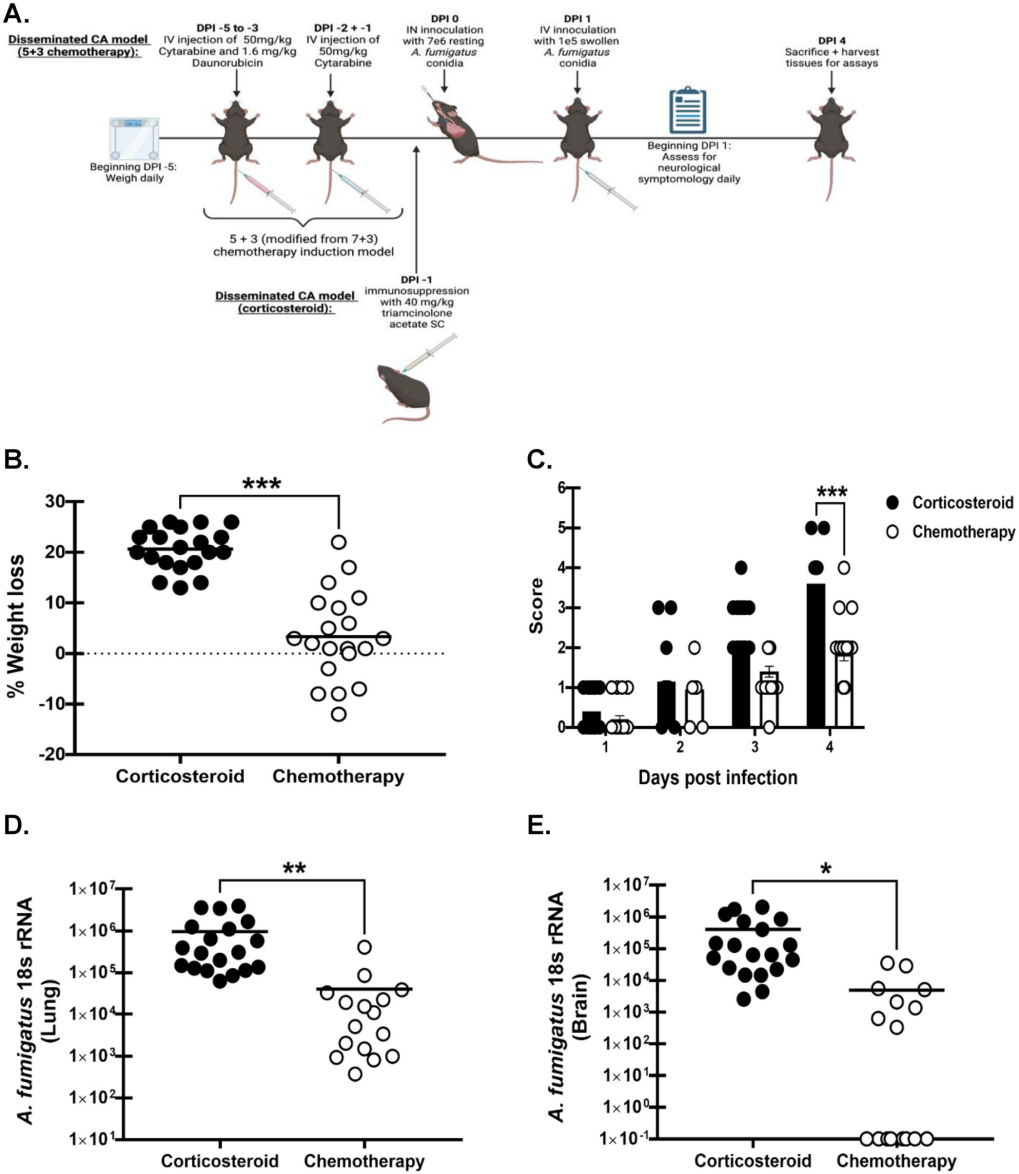
Comparison between corticosteroid-mediated immunosuppression and chemotherapy-mediated immunosuppression in the “two-hit” disseminated cerebral aspergillosis model. Mice were immunosuppressed with corticosteroids and exposed to A. fumigatus as in Fig. 1. (A) Mice were administered cytarabine (50 mg/kg) and daunorubicin (1.6 mg/kg) IV daily for 3 days and then cytarabine alone (50 mg/kg) IV should be replaced with “IV” for two additional days prior to A. fumigatus challenge, as in Fig. 1. Image created by BioRender.com. Mice were weighed daily beginning on day −5 to account for any effects of chemotherapy on weight loss. (B) Total percentage of weight loss was calculated by comparing mouse weight at the study endpoint (day 4) with that at the study initiation (day −1). (C) Beginning day 1, mice were assessed for disease severity and assigned a score between 0 and 5 correlating to the signs of clinical disease. (D) Lung and (E) brain fungal burdens on day 4 were assessed by RT-qPCR analysis of A. fumigatus 18S rRNA levels. Cumulative data from four independent studies. For all graphs, *, **, and *** represent *P* values of < 0.05, <0.01, and <0.001, respectively (unpaired two-tailed Student’s *t* test). *n* = 4 to 5 mice/group for each study; each data point represents a single mouse; line in each group corresponds to the mean. Panels B, D, and E: unpaired two-tailed Student’s *t* test. Panel C: two-way ANOVA (mixed effects) with *post hoc* Tukey’s multiple-comparison test.

In a recent systematic review, we identified acute myeloid leukemia (AML) as the primary hematologic malignancy associated with CA disseminating from IPA (27). The induction chemotherapy often employed in AML is a “7 + 3” regimen with the chemotherapeutic drugs cytarabine and daunorubicin (both drugs administered for the first 3 days, with cytarabine alone given for an additional 4 days) (28). In animal studies, a modified “5 + 3” regimen is employed; thus, we compared the “5 + 3” immunosuppressive strategy with the corticosteroid-mediated “two-hit” disseminated cerebral aspergillosis model (Fig. 6A) (29, 30). Weight was recorded daily beginning 5 days before infection to account for weight loss associated with the “5 + 3” regimen. Mice were monitored for clinical signs beginning on day 1 after infection. Mice included in the corticosteroid disseminated CA group (IN + IV + steroid) lost a significantly greater percentage of total body weight compared to the chemotherapy disseminated CA mice (IN + IV + chemo) (Fig. 6B). Disease severity was also significantly greater in the corticosteroid disseminated CA group (IN + IV + steroid) compared to the chemotherapy disseminated CA group (IN + IV + chemo) (Fig. 6C). In turn, corticosteroid-treated mice had higher lung fungal burdens than chemotherapy-treated mice (Fig. 6D). Thus, despite a known association with both corticosteroids and the “7 + 3” regimen for CA dissemination from IPA, corticosteroid-mediated immunosuppression was superior, with 100% of mice in this group having fungal burdens in the brain compared to only 50% of chemotherapy-treated mice.

### Comparison between corticosteroid-mediated immunosuppression and neutropenia in the “two-hit” disseminated cerebral aspergillosis model.

**FIG 7:**
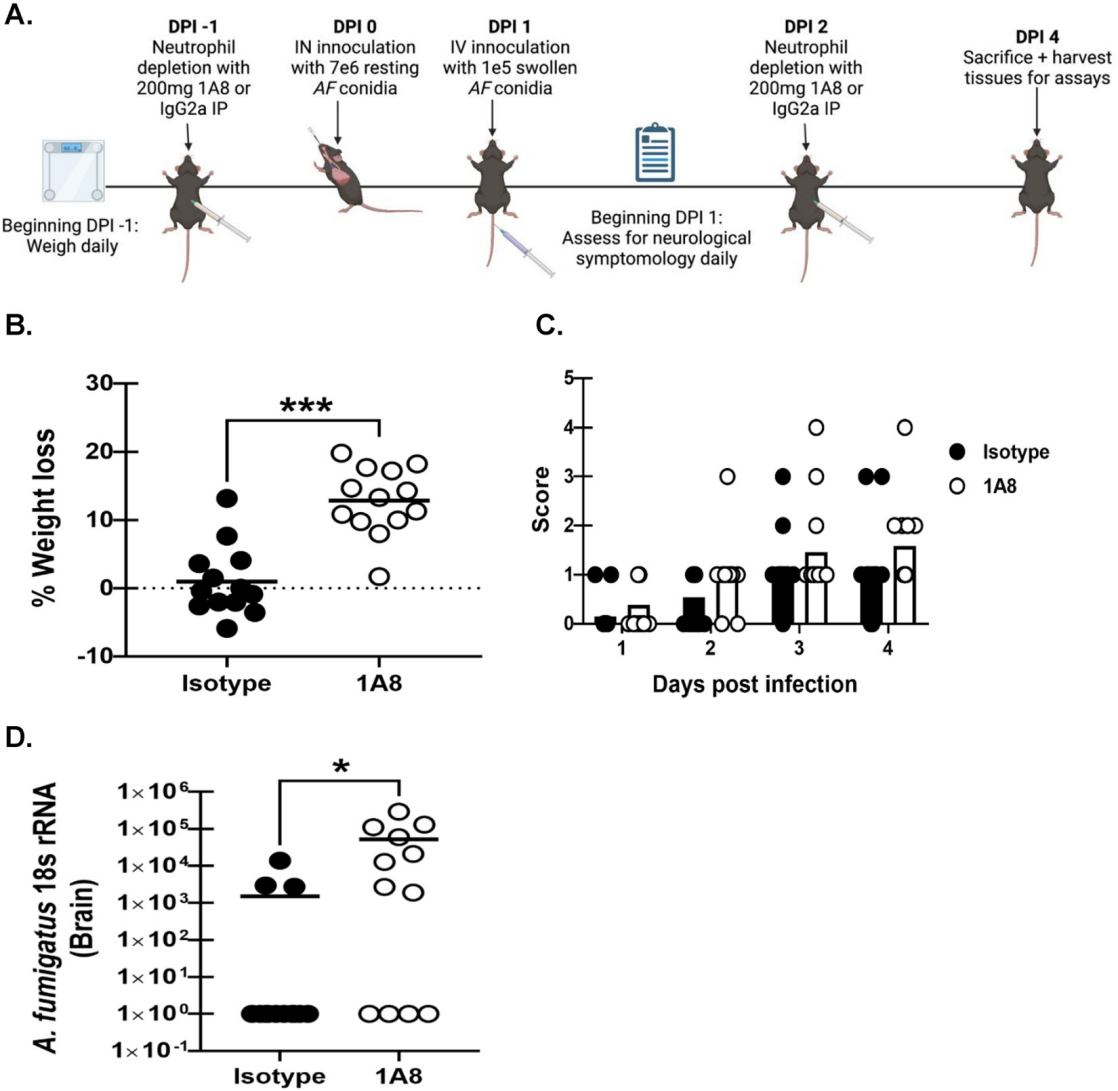
Comparison between corticosteroid-mediated immunosuppression and neutropenia in the “two-hit” disseminated cerebral aspergillosis model. Mice were immunosuppressed with corticosteroids and exposed to A. fumigatus as in Fig. 1. (A) Mice were subjected to neutropenia by administration of 200 μg anti-mouse Ly6G antibody (1A8) intraperitoneally (IP) 1 day prior to challenge with A. fumigatus as in Fig. 1 and again on day 3. Control mice were administered 200 μg IgG2a. Mice were weighed daily from study initiation (day −1) to study completion. Image created with BioRender.com. Following initial infection (day 1), mice were assessed for signs of disease severity daily and assigned scores ranging from 0 to 5. (B) Total percentage of weight loss was calculated by comparing mouse weight at the study endpoint (day 4) with that prior to immunosuppression (day −1). (C) Beginning day 1, mice were assessed for disease severity and assigned scores between 0 and 5 correlating to the signs of clinical disease. (D) Brain fungal burdens on day 4 were assessed by RT-qPCR analysis of A. fumigatus 18S rRNA levels. Cumulative data from three independent studies. For all graphs, * and *** represent *P* values of 0.05 and < 0.001, respectively (unpaired two-tailed Student’s *t* test) *n* = 4 to 6 mice/group for each study; each data point represents a single mouse; line in each group corresponds to the mean. Panels B and D: unpaired two-tailed Student’s *t* test. Panel C: two-way ANOVA (mixed effects) with *post hoc* Tukey’s multiple-comparison test.

Neutropenia is the primary risk factor for the development of IPA (31, 32). However, we were surprised to observe no differences in brain neutrophil numbers between the IN + IV and IN + IV + steroid groups, despite dramatic differences in fungal burden. Mice were administered neutrophil-depleting antibody or isotype control antibody and subjected to the “two-hit” disseminated cerebral aspergillosis model (Fig. 7A). Weight was recorded daily beginning at 1 day preceding infection and mice were monitored for clinical signs beginning day 1 after infection. Results showed that neutropenic mice (IN + IV + neutropenia) lost a significantly greater percentage of total body weight in comparison to the isotype control group (IN + IV+ Iso) (Fig. 7B). Disease severity was not significantly different between the two groups; however, some IN + IV + 1A8 mice did display signs of severe disease on day 4 (Fig. 7C). Neutropenia did, however, result in a higher fungal burden in the brain (Fig. 7D), although 33% of neutropenic mice did not have detectable A. fumigatus (compared to 77% of non-neutropenic mice). Thus, although neutropenia was associated with an increased presence of A. fumigatus in the brain, this was less consistent than in corticosteroid-treated mice.

## DISCUSSION

IPA is a disease characterized by uncontrolled growth of the opportunistic mold A. fumigatus, resulting in lung tissue damage and potential dissemination to other organs. The dominant patient populations at risk for developing IPA and subsequent mortality include those with leukemia or lymphoma, especially if these individuals undergo hematopoietic stem cell transplantation along with immunosuppression (33, 34). Of these, IPA is a significant concern in patients with AML (3, 34). On average, 20 to 50% of IPA infections result in disseminated disease, with ~20% reported to result in CA (3, 4). However, this is likely a conservative estimate as the population of CA is greatly underreported, partially due to the fact that CA is notably difficult to diagnose, with some cases not being diagnosed until autopsy (4, 35). Further, as the number of immunosuppressed patients continues to increase, the CA population is likely to be larger than reported (33, 35).

Recently, the use of corticosteroids, alone and in conjunction with therapeutics resulting in neutropenia, has been regarded as one of the most notable risk factors of IPA, given their frequent use in transplant recipients and patients with various HMs (9, 15). For example, in a post-transplant epidemiological study, corticosteroid use was a significant risk factor for IPA, as 87.5% of bone marrow transplant recipients diagnosed with IPA had been treated with corticosteroids (36). Similarly, a 20-year retrospective study of CA in patients with various underlying conditions found corticosteroids to be among the top risk factors, with more than 50% of patients with CA having undergone steroid treatment (9). During IPA, upon reaching the alveoli, A. fumigatus conidia swell and germinate, producing hyphae, all forms of which enter the pulmonary parenchyma (37, 38). A. fumigatus swollen conidia and hyphae invade the blood vessels through the endothelial cell lining by passing from the abluminal to the luminal surface, whereby swollen conidia and hyphal fragments can break off and disseminate through the bloodstream (37, 38). In the profoundly immunosuppressed host, invasion of the pulmonary vasculature can result in widespread hematogenous dissemination to organs such as the brain, kidneys, heart, spleen, and liver (35).

One limitation in understanding the development of, pathogenicity of, and immune responses during CA is the lack of an experimental animal model. A review of literature examining the development of or models for CNS/brain-associated CA identified a diverse array of models, yet none of these exactly replicate acquisition of IPA/CA as it relates to hematologic malignancies. Early models employed cyclophosphamide for immunosuppression and direct intracranial inoculation of A. fumigatus, which clearly resulted in CA, albeit this model does not replicate natural dissemination of the organism from the respiratory tract to the brain (12, 13). Other models employed cyclophosphamide for immunosuppression and intravenous inoculation; again, a model that results in A. fumigatus in the CNS/brain but again lacks the dissemination from the lung component (14, 39). Some studies have employed the neutrophil depleting antibody 1A8 with an intravenous challenge, or intravenous challenge without any immunosuppression (40–42). We found only a single study that employed a relevant cyclophosphamide-cortisone acetate immunosuppression model and administered A. fumigatus intratracheally, which resulted in 100% mortality by day 5 (43). Although live organism was detected in the lung in 100% of these mice, only 50% had detectable A. fumigatus in the brain (43).

Acknowledging the predisposing quality of corticosteroids for IPA and CA, the limitations of current animal models and the fact that entry of A. fumigatus resting conidia into the bloodstream is highly improbable, we developed a “two-hit” model of infection incorporating (i) a relevant immunosuppression strategy known to be associated with CA development in humans (corticosteroid therapy) (ii), a lung component (intranasal inoculation of resting conidia), and (iii) a dissemination component (intravenous inoculation of swollen conidia into the bloodstream). Although we recognize that employing intravenous inoculation is suboptimal, we felt that a combination of the above strategy had the best chance to mimic the pathogenesis of human disease and more effectively produce a predictable infection within the brain. Through this immunosuppression-based “two-hit” disseminated CA model (IN + IV + steroid), successful primary IPA and secondary disseminated CA was achieved, allowing for the characterization of pathogenicity and the neuroimmune response. In addition to severe weight loss and signs of severe disease, we observed fungal burdens in the brains of 100% of the mice and pathology consistent with that observed in the brains of humans with CA (17, 18). Moreover, mortality in the immunosuppression-based “two-hit” disseminated CA model was high, which also mimics that observed in humans with CA (5, 7, 44). In the brains of immunocompetent mice (IN + IV), we observed the infiltration of monocytes, eosinophils, DCs, CD4 T cells, CD8 T cells, iNKT cells, γδ T cells, and NK cells, all of which were at significantly lower levels in IN + IV + steroid mice. Intriguingly, no differences in macrophages or neutrophils were observed between immunocompetent and IN + IV + steroid mice. Given the significantly elevated fungal burdens in the brains of IN + IV + steroid mice, we can speculate that the recruitment or activation of one or more of the above cell types is negatively affected by corticosteroids. In contrast, IN + IV + steroid mice had significant infiltration of monocytes, eosinophils, macrophages, neutrophils, and NK cells compared to IN + steroid mice without CA, suggesting that the presence of A. fumigatus in the brain induces infiltration of certain leukocytes, regardless of immune status, even if it is not sufficient for clearing the fungal infection.

Many cytokines and chemokines are known to play essential roles in anti-fungal immunity during IPA (19, 45, 46). Our analysis revealed that IL-1β, IL-6, IL-17A, CCL3, CXCL10, and VEGF were decreased in the IN + IV + steroid group despite the presence of higher fungal burdens. In contrast, IL-1α, CXCL1, G-CSF, and eotaxin were increased in the IN + IV + steroid group. It could be argued that mediators which are lower in the IN + IV + steroid group are important in CNS antifungal defense. However, the decreased levels of these mediators may be a result of corticosteroid-mediated suppression. Alternatively, it could be argued that mediators which are higher in the IN + IV + steroid group are important in CNS antifungal defense, as these were resistant to corticosteroid-mediated suppression. However, the increased levels of these mediators may simply reflect the increased fungal burden in these mice. Nevertheless, we have made some compelling findings. Specifically, previous studies in the lung have shown that IL-1α, which is constitutively expressed, was essential for initiating leukocyte recruitment, while IL-1β, which is predominantly expressed under inflammatory conditions, was determined to be a critical mediator of antifungal activity against A. fumigatus hyphae (47). Interestingly, in the IN + IV + steroid mice, there was an imbalance between IL-1α and IL-1β in the brain, with IL-1α significantly elevated and IL-1β significantly decreased. Because IL-1β expression has been reported to be significantly impacted by corticosteroids, we can speculate here that corticosteroid immunosuppression during CA blunts antifungal activity within the brain by inhibiting IL-1β (48). During IPA, IL-6 is critically involved in the phagocytic activity of lung leukocytes, the absence of which results in increased fungal burdens in the lungs and decreased survival (49). Further, IL-6 is known to be a potent regulator of macrophage and DC differentiation from monocytes (50). Production of CXCL10 by neutrophils has likewise been identified as a critical mediator of DC recruitment to the lung during IPA (51). IL-6 and CXCL10 levels were significantly lower in IN + IV + steroid mice. Macrophage and DC recruitment into the brain during CA was blunted in IN + IV + steroid mice, and this may have been a result of the reduced production of IL-6 and CXCL10, respectively. Mice deficient in CCL3 are more susceptible to disseminated aspergillosis, whereas neutralization of CCL3 in neutropenic mice results in decreased survival and increased A. fumigatus burdens in the lung (52, 53). Blockage of the receptors for CXCL1 and CXCR2 results in increased mortality, higher A. fumigatus lung burdens, and lower neutrophil recruitment to the lung during IPA (54). Likewise, we have reported that the levels of CCL3 and CXCL1 directly correlate with neutrophils levels in the lung during IPA (55–57). Similar to IL-1α and IL-1β, there was an imbalance between CCL3 and CXCL1, with CCL3 being decreased in the IN + IV + steroid group and CXCL1 being increased. This may explain why neutrophils were not significantly different in IN + IV + steroid mice compared to IN + IV mice, despite the significant elevation in fungal burden in IN + IV + steroid mice. Indeed, CCL3 release from macrophages has been previously found to be downregulated following exposure to corticosteroids, whereas CXCL1 is seemingly unaffected by corticosteroid exposure (58, 59). IL-17A is a critical cytokine for both mediating neutrophil-mediated immunity and activating the antimicrobial response of epithelial cells (60–62). We have previously reported that IL-17A functions as a critical antifungal cytokine during IPA, with its levels directly correlating with the level of A. fumigatus lung burden but not necessarily with neutrophil levels (55, 56, 63–65). Although IL-17A is thought to be immunopathogenic in the brain during such diseases as experimental autoimmune encephalomyelitis, its role in CNS infections is not well described (66). However, acknowledging the importance of IL-17A in IPA and the observation of lower IL-17A in the brains of IN + IV + steroid mice, we cannot exclude a protective role for IL-17A during CA.

The lone cytokine that was reduced in IN + IV + steroid mice, compared to that in both IN + IV and IN + steroid mice, was VEGF. VEGF is a proangiogenic mediator which may be modulated by A. fumigatus gliotoxin and other secondary metabolites (67). In a previous study employing an immunosuppression murine model of IPA, treatment with recombinant VEGF prolonged survival, although it did not reduce fungal burden (68). Additionally, to date, several cases of IPA in cancer patients treated with the new anti-cancer therapeutics bevacizumab, a VEGF inhibitor, or pazopanib, a VEGF receptor inhibitor, have been reported (69, 70). These results suggest that targeting angiogenesis may potentially be protective against the incidence of CA. However, IN + IV + steroid mice, daily VEGF administration for 4 days had no impact on the brain fungal burden on day 4 (Fig. S2). Thus, even though VEGF is reduced in IN + IV + steroid mice with a high brain fungal burden, VEGF treatment alone is not sufficient to improve the outcome of “two-hit” disseminated CA.

10.1128/mbio.02254-22.2 FIG S2 Effects of VEGF treatment in an immunosuppression-based “two-hit” disseminated cerebral aspergillosis model. Mice were immunosuppressed with corticosteroids and exposed to A. fumigatus as in Fig. 1. (A) Mice were randomized to receive recombinant mouse vascular endothelial growth factor 164 (rm-VEGF_164_, 200 ng in 100 μL), which was administered subcutaneously (SC), daily from days post infection 0 to 3 for a total of 4 doses (R&D Systems, Minneapolis, MN). Control mice received phosphate-buffered saline + 0.1% bovine serum albumin, sterile-filtered in 100 μL. Image created by BioRender.com. (B) Total percentage of weight loss was calculated by comparing mouse weight at the study endpoint (day 4) with that at the study initiation (day −1). (C) Beginning day 1, mice were assessed for disease severity and assigned scores between 0 to 5 correlating to signs of clinical disease. (D) Brain fungal burden at day 4 was assessed by reverse transcription-quantitative PCR analysis of A. fumigatus 18S rRNA levels. The Figures illustrate cumulative data from two independent studies, *n* = 5 mice/group for each study; each data point represents a single mouse and the center line in each group corresponds to the mean. Data were analyzed using GraphPad Prism version 9.0 statistical software (GraphPad Software, San Diego, CA). Panels B, D, and E: unpaired two-tailed Student’s *t* test. Panel C: two-way analysis of variance (mixed effects) with *post hoc* Tukey’s multiple-comparison test. Download FIG S2, TIF file, 0.3 MB. Copyright © 2022 Sullivan et al. 2022 Sullivan et al. https://creativecommons.org/licenses/by/4.0/ This content is distributed under the terms of the Creative Commons Attribution 4.0 International license.

Neutropenia has historically been considered the predominant risk factor for IPA, particularly for HM patients undergoing induction chemotherapy (71). As stated earlier, AML is the primary HM at risk for IPA. AML patients often receive a “7 + 3” induction chemotherapy regimen which involves treatment with two chemotherapy drugs, cytarabine and an anthracycline drug such as daunorubicin, with the resulting neutropenia often associated with the development of infection (72, 73). Although these chemotherapeutic agents are extensively used in AML and there have been numerous reports of aspergillosis occurring in patients administered these drugs, we found no reports that examined daunorubicin in an animal model and only a single report of a rabbit model that employed cytarabine + methylprednisolone for immunosuppression (74). Although we employed a similar “5 + 3” regimen in mice which used human-equivalent dosing to effectively mimic the immunosuppressive state frequently associated with A. fumigatus infection, development of disseminated CA occurred at lower levels in terms of fungal burden in the brain and in fewer mice compared to mice immunosuppressed with corticosteroids (IN + IV + steroid) (29, 30). Although depletion of neutrophils with antibodies is artificial in terms of human disease and does not represent how neutrophil depletion actually occurs as a result of chemotherapy regimens, to more specifically evaluate neutropenia-based immunosuppression for modeling disseminated CA, we used an IN + IV model with the neutrophil depleting antibody 1A8. Induction of neutropenia was less efficient at producing disseminated CA compared to corticosteroid-induced immunosuppression in mice. Although data with 1A8 do overall support a role for neutrophils in limiting A. fumigatus, it is not clear whether the lack of neutrophils is required to control it in the brain or the lack of neutrophils results in its increased escape from the lung.

In summary, we have developed a human-relevant, novel model of disseminated CA by utilizing corticosteroid-induced immunosuppression and a “two-hit” inoculation method with A. fumigatus. With this model, we achieved severe clinical disease, 100% mortality, and significant fungal burdens in both the lung and the brain in 100% of the mice. Furthermore, we found that corticosteroid-induced immunosuppression significantly blunted infiltration of specific immune cells, as well as inflammatory cytokine and chemokine responses, in the brain during CA. Finally, we demonstrated that corticosteroid-induced immunosuppression is superior at inducing disseminated CA to chemotherapy-induced or neutropenia-induced immunosuppression. In conclusion, this model of disseminated CA following IPA in an immunosuppressed host provides a novel platform for studying the efficacy of antifungal drugs and immunotherapies for improving disease outcomes.

## MATERIALS AND METHODS

### Mice.

Male and female age-matched C57BL/6 mice, 7 to 10 weeks of age, were obtained from The Jackson Laboratory (Bangor, ME). All animals were housed in a specific pathogen-free, Association for Assessment and Accreditation of Laboratory Animal Care-certified facility, and handled according to Public Health Service Office of Laboratory Animal Welfare policies after review by the Tulane Institutional Animal Care and Use Committee (IACUC). All animal research was conducted under the approved Tulane IACUC Protocol no. 1589. Mice were euthanized following anesthesia with ketamine/xylazine (100/10 mg · kg^−1^ intraperitoneally [IP]; MWI Veterinary Supply, Boise, ID). No animals were excluded from the analyses unless they died prematurely. The letter ‘*n*’ as shown in the manuscript represents the number of animals in each group that were euthanized as scheduled at the end of the study unless otherwise stated.

### Preparation of *A. fumigatus*.

A. fumigatus isolate 13073 (American Type Culture Collection, Manassas, VA) was maintained on potato dextrose agar for 5 to 7 days at 37°C. Conidia were harvested by washing the culture flask with 50 mL of sterile phosphate-buffered saline (PBS; Thermo Fisher Scientific, Waltham, MA) supplemented with 0.1% Tween 20 (Bio-Rad, Hercules, CA). The conidia were then passed through a sterile 40-mm nylon membrane to remove hyphal fragments and conidial clusters and enumerated on a hemacytometer.

### Immunosuppression.

For the corticosteroid-induced immunosuppression model, mice were given Kenalog-40 or sterile PBS as an immunocompetent control (75). Briefly, subcutaneous (SC) injections of 40 mg Kenalog-40 (triamcinolone acetonide; Bristol-Myer Squibb, Princeton, NJ) per kg body weight in sterile PBS for a final volume of 100 μL were administered 24 h before fungal inoculation (76). Immunocompetent mice were injected SC with 100 μL of sterile PBS 24 h prior to fungal inoculation. For the 5 + 3 chemotherapy-induced immunosuppression, mice were given a regimen of cytarabine (Hospira, Lake Forest, IL) and daunorubicin HCl (Selleck Chemicals, Houston, TX) as previously described (29, 30). Briefly, intravenous injections of 50 mg/kg cytarabine and 1.6 mg/kg daunorubicin in sterile PBS for a final volume of 100 μL were administered daily for 3 days beginning 5 days prior to fungal inoculation. Two days prior to fungal inoculation, mice were weighed and given daily IV injections of 50 mg/kg cytarabine in sterile PBS for a final volume of 100 μL. For neutrophil depletion, mice were given anti-mouse Ly6G antibody (clone 1A8; BioXcel, West Lebanon, NH) to induce neutropenia or isotype IgG2a (clone 2A3; BioXcel) for the nonneutropenic control as previously described (77, 78). Briefly, IP injections of 200 μg 1A8 or IgG2a in sterile PBS for a final volume of 100 μL were administered 1 day before intranasal (IN) fungal inoculation and 1 day following IV fungal inoculation to ensure continuous neutropenia for the duration of the experiment.

### Infection.

For pulmonary infection, mice were lightly anesthetized with isoflurane and administered 7 × 10^6^ A. fumigatus conidia in a volume of 30 μL IN at 1 day post-immunosuppression, as previously described (79). Briefly, mice were held in a horizontal, supine position, and a pipette was used to deliver the 30-μL inoculum dropwise to the nares, where normal breathing results in fluid aspiration into the lungs. For disseminated infection, 1 day following IN inoculation, mice were administered 1 × 10^5^ A. fumigatus swollen conidia (cultured for 5 h at 37°C) in a volume of 100 μL delivered via IV injection through the lateral tail vein (80). IN-only control mice were IV administered 100 μL of sterile PBS, incubated at 37°C for 5 h, day post-IN inoculation.

### Monitoring disease severity.

Mice were weighed daily and the percentage of total weight loss between study initiation and completion was calculated. Following infection, mice were scored daily beginning 1 day post-IN inoculation for morbidity and mortality for up to 10 days, using a modified scoring system as previously described (81). Morbidity was scored from 0 to 5 as follows: 0, healthy; 1, minimal disease (e.g., ruffled fur); 2, moderate disease (e.g., ungroomed, hunched); 3, severe disease (e.g., severely hunched, altered gait, low motility, head-tilt); 4, moribund (e.g., spinning in cage or when suspended by tail, unable to move freely around cage, >25% weight loss); and 5, deceased. Mice which received a score of 4 were humanely euthanized.

### Survival studies.

Cages were inspected 2 to 3× daily. All animals were observed for signs of disease severity, weight loss, and morbidity for a total of 10 days after initial IN infection. Morbidity was recorded for mice that reached a score of 4 or 5. Mice which survived to day 10 were euthanized.

### Lung and brain fungal burden assessment.

For lung fungal burden analysis, the left lung was collected on day 4 postinduction and homogenized in 1 mL PBS. Total RNA was extracted from 0.1 mL of unclarified lung homogenate using the MasterPure Yeast RNA purification kit (Lucigen Corporation, Middletown, WI), which includes a DNase treatment step to eliminate genomic DNA, as previously reported, because DNA is not predictive of organism viability in this assay (82). For brain fungal burden analysis, the right hemisphere was collected at day 4 and homogenized in 0.6 mL PBS. Total RNA was extracted in the same manner as for the lung from 0.3 mL of unclarified brain homogenate using the MasterPure Yeast RNA purification kit, with the exception of extraction reagent and proteinase K volumes, which were doubled. Lung and brain A. fumigatus burdens were analyzed using reverse transcription-quantitative PCR (RT-qPCR) measurement of A. fumigatus 18S rRNA (Integrated DNA Technologies; forward, 5′-GGC CCT TAA ATA GCC CGG T-3′; reverse, 5′-TGA GCC GAT AGT CCC CCT AA-3′; probe, 5′-/56-FAM/AGC CAG CGG CCC GCA AAT G/3BHQ_1/-3′) and quantified using a standard curve of A. fumigatus 18S rRNA synthesized by GenScript via *in vitro* transcription. The final RNA sequence of the 18S fragment (562 nt) is as follows: TCGCAAGGCTGAAACTTAAAGAAATTGACGGAAGGGCACCACAAGGCGTGGAGCCTGCGGCTTAATTTGACTCAACACGGGGAAACTCACCAGGTCCAGACAAAATAAGGATTGACAGATTGAGAGCTCTTTCTTGATCTTTTGGATGGTGGTGCATGGCCGTTCTTAGTTGGTGGAGTGATTTGTCTGCTTAATTGCGATAACGAACGAGACCTCGGCCCTTAAATAGCCCGGTCCGCATTTGCGGGCCGCTGGCTTCTTAGGGGGACTATCGGCTCAAGCCGATGGAAGTGCGCGGCAATAACAGGTCTGTGATGCCCTTAGATGTTCTGGGCCGCACGCGCGCTACACTGACAGGGCCAGCGAGTACATCACCTTGGCCGAGAGGTCTGGGTAATCTTGTTAAACCCTGTCGTGCTGGGGATAGAGCATTGCAATTATTGCTCTTCAACGAGGAATGCCTAGTAGGCACGAGTCATCAGCTCGTGCCGATTACGTCCCTGCCCTTTGTACACACCGCCCGTCGCTACTACCGATTGAATGGCTCGGTGAGGCCTTCGGA. RT-qPCR was performed using the CFX96 Real-Time System C1000 Touch thermal cycler (Bio-Rad).

### Inflammatory cytokine analysis.

Following euthanasia at day 4, the right hemisphere of the brain was flash-frozen and stored at −80°C until processing. The flash-frozen brain tissue was homogenized as previously described, with minor modifications (83). Briefly, brain tissue was homogenized in a buffer containing 20 mmol/L Tris-HCl (pH 7.5) (Thermo Fisher Scientific), 150 mmol/L NaCl (Thermo Fisher Scientific), 0.05% Tween 20, a cocktail of protease inhibitors (Roche, Basel, Switzerland), and freshly added 1 mmol/L phenylmethylsulfonyl fluoride (Roche). The homogenate was sonicated for 30 s and clarified by centrifugation (12,000 × *g* for 10 min at 4°C). Clarified brain homogenate supernatants were analyzed for the protein levels of 32 inflammatory cytokines and chemokines using the Luminex-based Milliplex multiplex suspension cytokine array (Millipore Sigma, Burlington, MA), according to the manufacturer’s instructions. The data were analyzed using Bio-Plex Manager software (Bio-Rad).

### Flow cytometry.

Following euthanasia at day 4, whole brains were extracted and mononuclear cells were dissociated from CNS tissue, separated on a density gradient, blocked for nonspecific antibody binding, and stained as previously described, with minor modification (84). Brain tissues from individual animals were minced gently in RPMI 1640 medium (Thermo Fisher Scientific) with scissors and incubated with collagenase D (1 mg/mL; Roche) and DNase I (50 μg/mL; Roche) for 30 min in a shaking incubator at 37°C. Digested tissues were passed through a 100-μm cell strainer (Corning, Corning, NY) to remove cell debris and obtain single-cell suspensions. Cells were spun down at 256 × *g* for 5 min at 4°C. The resultant cell pellets from brain tissues were resuspended in ice-cold 90% Percoll PLUS solution (Cytiva, Marlborough, MA) and overlaid with 60% Percoll PLUS solution, followed by 40% Percoll PLUS solution and subsequently by 1× Hanks balanced salt solution (HBSS; Thermo Fisher Scientific) gently. All Percoll solutions were diluted in 1× HBSS. Cells were isolated by centrifugation at 514 × *g* for 20 min at room temperature with the brake disengaged. The cell fraction (containing mononuclear cells) located between the 60% and 40% interphase was carefully aspirated. Cell suspensions were washed with 1× HBSS and centrifuged at 348 × *g* for 10 min at 4°C. The resultant cell pellets were resuspended in 500 μL FACS buffer (PBS + 2% bovine serum albumin [BSA] + 1mM EDTA + 0.09% sodium azide). Cells were washed, and Fc receptors were blocked with Mouse TruStain FcX (CD16/32; BioLegend, San Diego, CA) at 4°C for 20 min. After that, cells were stained with a single-color LIVE/DEAD Aqua fixable dead-cell stain (Invitrogen), then labeled with specific immune cell surface and intracellular markers. The following staining parameters were employed: microglia, CD45intCD11b^+^ TMEM119^+^; M1 microglia, CD45intCD11b^+^ CD86^+^ CD206^–^; M2 microglia, CD45intCD11b^+^ CD86^−^ CD206^+^; astrocytes, CD45^−^ CD11b^−^ ASCA-2^+^ A2B5+; reactive astrocytes, CD45^−^ CD11b-ASCA-2^+^ A2B5^+^ GFAP^+^; macrophages, CD45hiCD11b^+^ TMEM119^−^; eosinophils, CD45^+^ CD11b^+^ SiglecF^+^; neutrophils, CD45^+^ CD11b^+^ Ly6G^+^; monocytes, CD45^+^ CD11b^+^ CD11c^−^ Ly6C^+^; DCs, CD45^+^ CD11c^+^ Ly6C^+^ MHC II^+^; CD4^+^ T cells, CD45^+^ CD3^+^ CD4^+^; CD8^+^ T cells, CD45^+^ CD3^+^ CD8^+^; natural killer T cells (NKTs), CD45^+^ CD3^+^ CD1d^+^; γδ T cells, CD45^+^ CD3^+^ TCRgd^+^; NKs, CD45^+^ CD3^−^ NK1.1^+^. The specifics of antibodies used are detailed in Table S1. Samples were acquired using a 4-laser, 20-parameter analytic BDLSRFortessa, and data were analyzed using FlowJo software (Tree Star, Inc., Ashland, OR). Unstained brain leukocytes served as a control for background fluorescence and gating. Appropriately stained UltraComp eBeads (Thermo Fisher Scientific) served as single-color controls unless otherwise indicated.

10.1128/mbio.02254-22.3 TABLE S1 Antibodies for flow cytometry. Download Table S1, TIF file, 0.1 MB. Copyright © 2022 Sullivan et al. 2022 Sullivan et al. https://creativecommons.org/licenses/by/4.0/ This content is distributed under the terms of the Creative Commons Attribution 4.0 International license.

### Histology.

The left hemisphere of the brain was collected and fixed in 10% neutral buffered formalin. The fixed brains were paraffin-embedded and then processed and stained with H&E or GMS by GNO Histology Consultants (New Orleans, LA). Imaging was performed using a Swift Optical Instruments M10T-P Trinocular LED Microscope equipped with a Motic Moticam 5 + 5-megapixel digital camera.

### Statistics.

Data were analyzed using GraphPad Prism version 9.0 statistical software (GraphPad Software, San Diego, CA). Comparisons between groups for normally distributed data were made with a Student’s *t* test or two-way analysis of variance. Significance was set at *P* < 0.05.
