# Supplementary figures and images for: Neuroimmune Responses in a New Experimental Animal Model of Cerebral Aspergillosis
Source: mBio. 2022 Aug 30;13(5):e02254-22. doi: 10.1128/mbio.02254-22 (PMC9600342; doi:10.1128/mbio.02254-22)

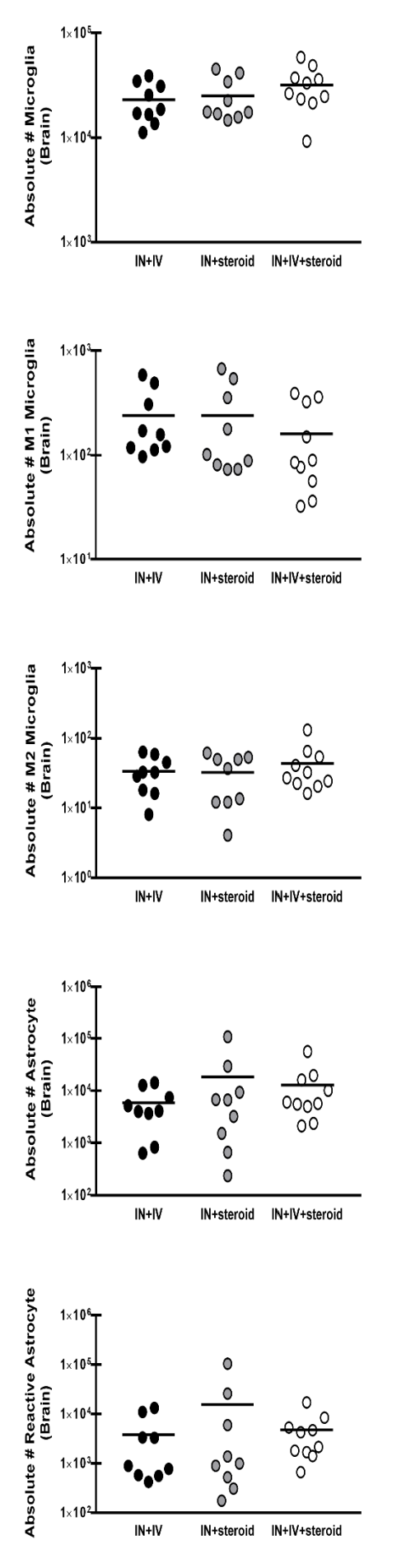

Supplement: FIG S1 [file mbio.02254-22-s0002.tif]

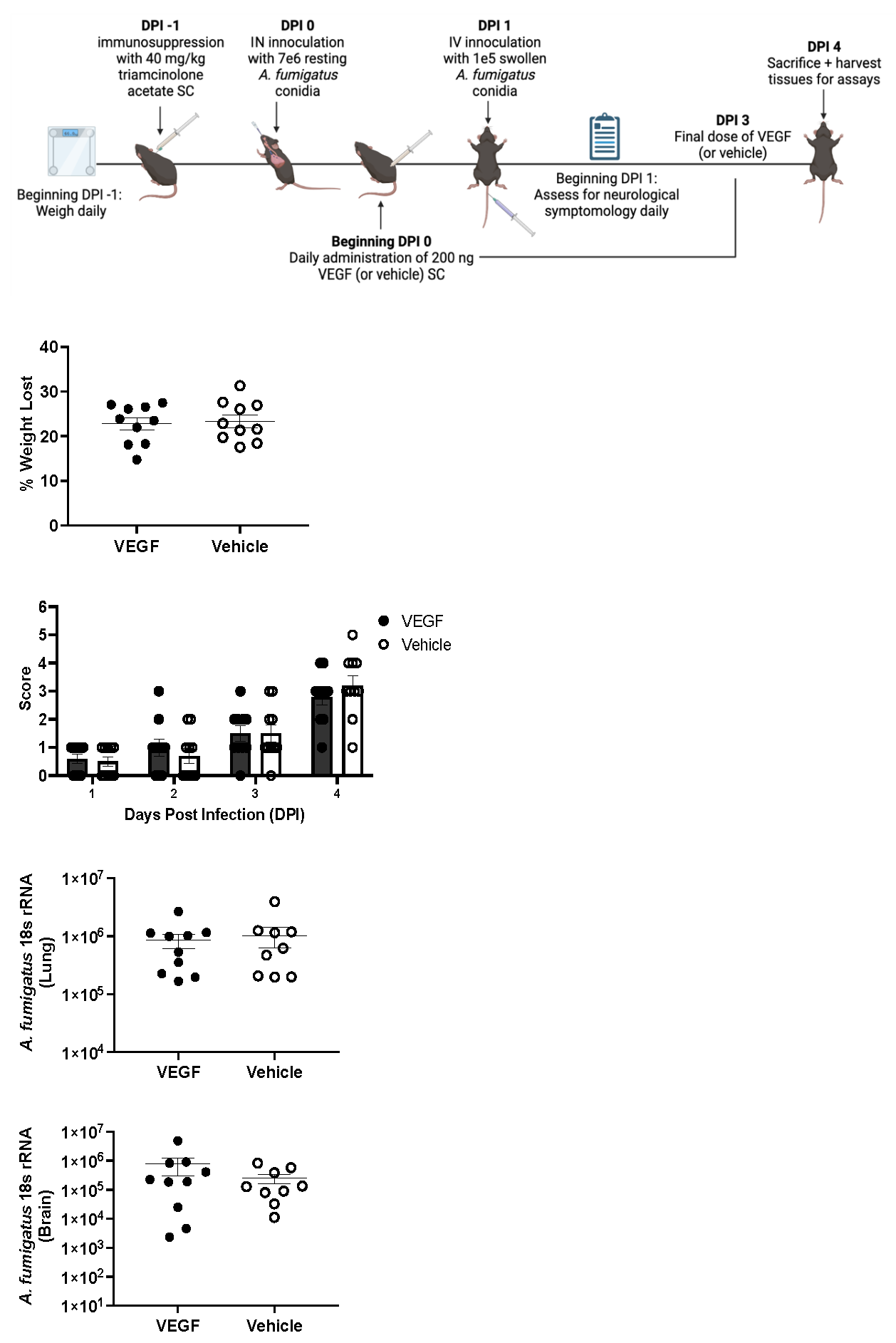

Supplement: FIG S2 [file mbio.02254-22-s0003.tif]

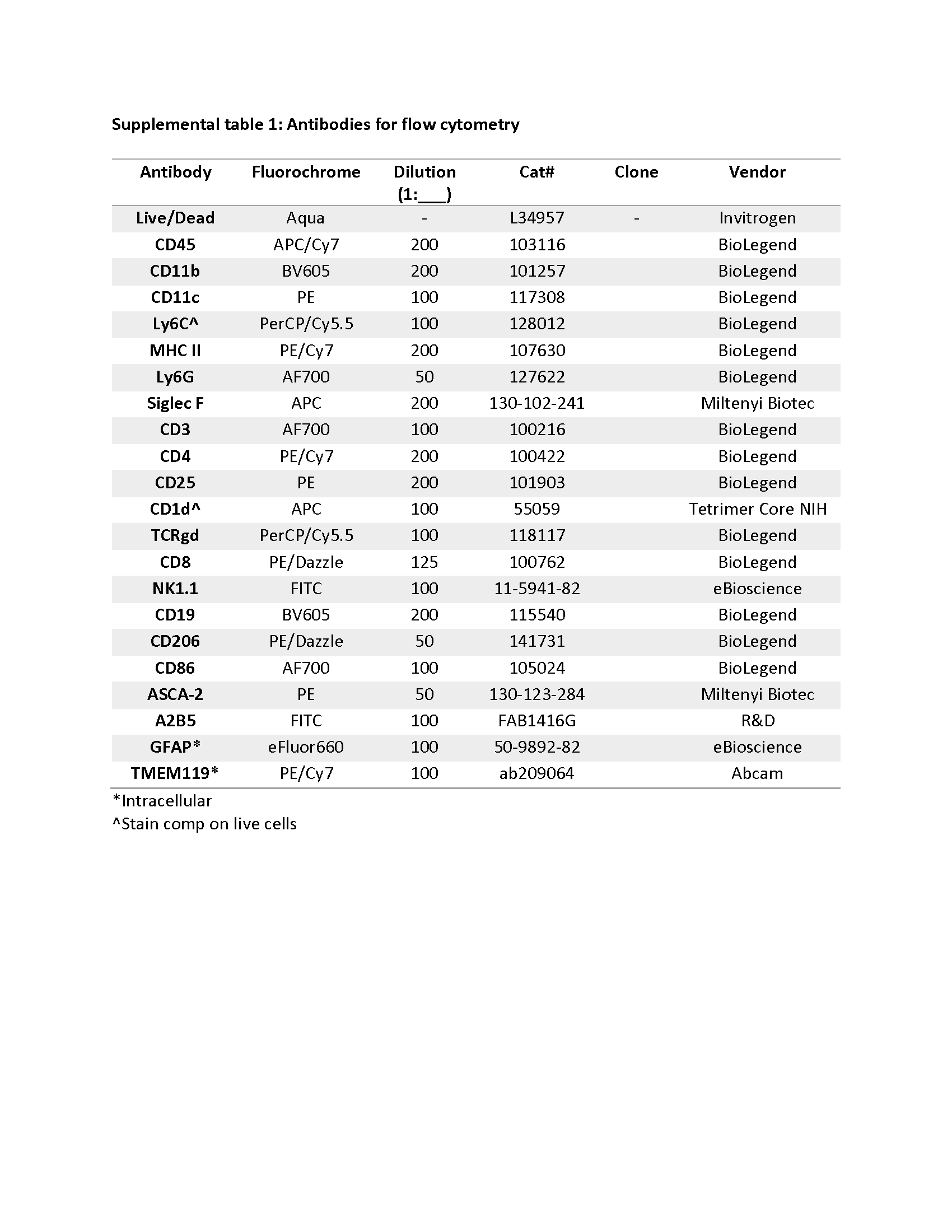

Supplement: TABLE S1 [file mbio.02254-22-s0001.tif]
